# Rickettsia retinitis cases in India: a few comments

**DOI:** 10.1186/s12348-016-0076-1

**Published:** 2016-02-27

**Authors:** Koushik Tripathy, Rohan Chawla, Yog Raj Sharma, Rajpal Vohra

**Affiliations:** Dr. Rajendra Prasad Centre for Ophthalmic Sciences, All India Institute of Medical Sciences, New Delhi, 110029 India

**Keywords:** Weil–Felix test, Optical coherence tomography, ELISA, PCR, Kyrieleis plaques

## Abstract

An important cause of infectious retinitis, not well-described in Indian literature, has been analyzed in detail systematically by Kawali A. and colleagues. However, Rickettsia retinitis (RR) was diagnosed at titres of 1:160 by the Weil–Felix test (WFT). The sensitivity and specificity of WFT at this level are poor compared to the gold standard immunofluorescent antibody assay. However, we understand that financial constraints of the Indian patients limit the availability of more definite tests. In our opinion, the optical coherence tomography features of RR described by the authors may be mimicked by other causes of retinitis, such as toxoplasma retinitis or even cotton wool spots. Infectious retinitis including RR should be treated by an antimicrobial agent with or without oral steroids until larger series or randomized controlled trials prove otherwise.

## Editor,

We read with interest the article by Kawali A. and colleagues [1]. The authors have described an important infectious retinitis which has not been evaluated systematically in peer-reviewed literature from India. We would like to humbly make the following comments:

1. The diagnosis of Rickettsial retinitis (RR) was based on the Weil–Felix test (WFT) at titres of 1:160 or more [1]. WFT at a titer of 1:320 has been shown to have poor sensitivity and specificity (46 %) when compared to the gold standard immunofluorescent antibody assay [2]. WFT may also be positive in the healthy population (54 %) and patients with non-rickettsial fever (62 %) [2]. The Indian Council of Medical Research guidelines [3] considers a WFT titre of 1:80 for probable rickettsial infection. A fourfold rise of titres in paired sera or a titre of 1:320 has been traditionally used as a diagnostic cutoff for WFT; however, baseline titres need standardization according to geographic location [4]. Using a WFT titre of 1:160 needs validation [1]. Also, more definite tests like IgM and IgG enzyme-linked immunosorbent assay (ELISA) and polymerase chain reaction (PCR) are available in India. However, we do understand that financial constraints of Indian patients are important barriers for the use of such tests.
2. We have seen hyperreflectivity of inner retinal layers in various types of retinitis, and this may not be a distinguishing feature of rickettsial retinitis as proposed by the authors. Retinitis due to other causes such as toxoplasma has been shown to involve the whole retina on optical coherence tomography (OCT) [5]. However, this may not always be the case. Hyperreflectivity of the inner retina with retinal thickening and shadowing may be a feature of cotton wool spots [5] or toxoplasmosis as well [6].
3. Kawali and coauthors propose [1] that “Once the patients are diagnosed as RR or presumed RR, they can be started on steroids and antibiotics.” We believe that for all infectious retinitis cases, the primary treatment should be the antimicrobial agent. Steroids can be started under antimicrobial cover if the intraocular inflammation component is severe. Though the three patients reportedly improved with steroids alone, Kawali A. et al. [1] acknowledge that all of them had taken antimicrobial agents during fever. In light of our current knowledge of cases of infectious retinitis and due to lack of a randomized trial comparing the effect of steroid or antimicrobial alone versus their combination, it may not be prudent to start a case of rickettsial retinitis on steroid alone without proper antibiotic cover. We believe that this may in fact worsen the condition.
4. Kyrieleis plaques have been described in *Rickettsia conorii* infection [7, 8] by Khairallah M. and colleagues. We would like to know from the authors if they found such plaques in any of their cases. We have described a case of Kyrieleis plaques in acute retinal necrosis [7] and do believe that they are seen in retinitis of various etiologies including active toxoplasma retinitis.
